# Crisis-focused psychosocial interventions for borderline personality disorder: systematic review and narrative synthesis

**DOI:** 10.1192/bjo.2022.54

**Published:** 2022-05-17

**Authors:** Lisa Wood, Liberty Newlove

**Affiliations:** Division of Psychiatry, University College London, UK; and Acute and Rehabilitation Directorate, North East London NHS Foundation Trust, London, UK; Acute and Rehabilitation Directorate, North East London NHS Foundation Trust, London, UK

**Keywords:** Personality disorder, systematic review, narrative synthesis, mental health crisis, psychosocial interventions

## Abstract

**Background:**

Mental health crisis presentations are common in those who have a diagnosis of borderline personality disorder (BPD), and psychosocial interventions should be provided. However, there is limited evidence outlining what a crisis-focused psychosocial intervention for this population should include.

**Aims:**

To conduct a systematic review and narrative synthesis of crisis-focused psychosocial interventions for people diagnosed with BPD.

**Method:**

Three databases (MEDLINE, Embase, PsycInfo) were searched for eligible studies. Studies were included if they were quantitative studies comparing a crisis-focused intervention with any control group and they included adults (18+ years of age) who were diagnosed with BPD (or with equivalent experiences). A narrative synthesis was undertaken to analyse results.

**Results:**

A total of 3711 papers were initially identified, 95 full texts were screened and 5 studies were included in the review. Two of five studies reported on the same trial, so four individual trials were included. Overall moderate risk of bias across studies was identified. The review tentatively demonstrated that crisis-focused psychosocial interventions are feasible and acceptable to people with BPD and that they have potential impacts on outcomes such as self-harm and number of days spent in hospital. There is limited consensus on what outcome measures should be used to assess the impact of interventions.

**Conclusions:**

There is presently insufficient data to recommend any specific psychosocial crisis intervention for people with BPD. Given the relationship between BPD and the high frequency of crises experienced by this group, further large-scale trials examining crisis-focused psychosocial interventions are required.

**Statement on language:**

We acknowledge that the term personality disorder can be controversial and stigmatising. As the term borderline personality disorder has been retained in DSM-5 and is used in the research evidence base we have decided to use this term throughout this review. However, we recognise that this term may not be acceptable to all.

Acute mental health crisis presentations span a range of symptomology, precipitating events and clinical diagnoses. A crisis can be defined as an event that the person perceives as threatening and is unable to reduce or manage, resulting in increased fear, confusion and extreme emotional distress.^1^ A significant proportion of individuals presenting in crisis to acute mental health services such as psychiatric hospitals and home treatment services have a diagnosis of borderline personality disorder (BPD).^2^ Research has highlighted that a diagnosis of BPD is associated with frequent crisis presentations and increased risk of self-harm and suicidality.^2,3^ Up to 10% of people with BPD die by suicide and the risk of this occurring increases with every suicide attempt.^4^ Moreover, 65–80% of people with BPD will engage in self-harming behaviour when in distress.^4^ Reducing self-harm and suicide and improving crisis care is a UK government priority outlined in the NHS Long Term Plan,^5^ as well as a wider international priority.^6^ The economic burden of BPD is also high: the direct annual care cost per patient has been estimated at £13 700 (€16 000) in a German setting, with 90% of this amount due to in-patient care.^7,8^ Therefore, preventing crisis and in-patient admissions has important economic benefits.

When an individual presents in crisis, it is likely to result in contact with acute mental health services such as home treatment teams, emergency departments and in-patient wards. Contact with acute services, particularly in-patient wards, has been shown to have iatrogenic effects and to increase the risk of self-harm and suicide in those with BPD.^9^ This is because these services do not meet the needs of this population, are not trauma-informed and may serve to re-traumatise individuals and reduce the likelihood of help-seeking in the future.^10,11^ Frequent presentation to crisis services is a concern for clinicians and policy makers alike. One of NHS England's priorities is to reduce the frequency of crisis presentations of those with complex needs, to reduce service pressures and improve the quality of care provided.^12^ However, we continue to lack effective strategies to do this.

It is well-known that people generally report being unhappy with the treatment they receive in crisis care. A recent systematic review of qualitative literature conducted by DeLeo et al^8^ identified four key themes: (a) acceptance and rejection when presenting to crisis care: limited options and lack of involvement of carers; (b) interpersonal processes: importance of the therapeutic relationship and establishing a framework for treatment; (c) managing recovery from a crisis: a clear recovery plan and negotiating collaboration; and (d) equipping and supporting staff: training and emotional support. These themes demonstrated that people with BPD often feel rejected by crisis services and that staff do not have the skills to work effectively with them. The review demonstrates that there is a clear lack of psychosocial interventions in this setting, which may explain this finding, and that further development of psychosocial interventions may be helpful in addressing these concerns.

A pragmatic review of nine National Institute for Health and Care Excellence (NICE) guidelines gave an overview of the effectiveness of existing crisis interventions across a range of clinical presentations, including BPD.^13^ It highlighted that there was a lack of randomised controlled trials (RCTs) and that the evidence for crisis interventions in general was of low quality. At the time of publication of the most recent NICE guidelines for BPD,^14^ no RCTs were found of psychological interventions for those with BPD experiencing crisis. There continues to be a lack of evidence to guide crisis care for those with BPD and a review conducted in 2012 failed to find a single RCT of a crisis-focused psychosocial intervention for BPD.^3^ There have been no systematic reviews of crisis interventions since that time, which would be important to shed light on the current evidence base and developments in this field.

Pharmacological interventions for this population have limited efficacy and there is no firm evidence that they help during an acute crisis.^14^ NICE recommends limited and cautious prescribing of psychiatric medication for BPD.^14^ Therefore, when managing a crisis, the focus is on the provision of psychosocial interventions.^14^ The limited evidence available has shown that stepped care using psychologically informed crisis interventions may be useful when a person with a diagnosis of BPD is in crisis, but there is no clear summary of the evidence. The present study aimed to conduct a systematic review and narrative synthesis of crisis-focused psychosocial interventions for people diagnosed with BPD. More specifically, it aimed to examine: the psychosocial interventions available, the quality of the evidence, the types of measures used to examine outcome, and the impact of the interventions on key outcomes (readmission to hospital, psychiatric symptoms, functioning, quality of life and days in hospital).

## Method

### Design

This study is a systematic review and narrative synthesis of available crisis-focused psychosocial interventions for people with BPD. We followed guidance from the Preferred Standards for Systematic Reviews and Meta-Analysis (PRISMA)^15^ and pre-registered the review protocol on PROSPERO (CRD42021268345).

### Study inclusion criteria

Studies were included if they: (a) were quantitative studies (including cohort studies, controlled studies and (cluster) randomised controlled trials) comparing a crisis-focused intervention with any control group; (b) included adults aged 18+ years who had a diagnosis of emotionally unstable personality disorder (EUPD) or BPD or met the threshold for BPD diagnosis (i.e. presented with complex emotional needs or complex trauma but did not have a formal diagnosis); and (c) examined a crisis-focused psychosocial intervention, defined as a non-pharmacological intervention targeting psychological or social factors. Studies were excluded if they were quantitative studies using a case–control, cross-sectional, case study, case series, qualitative or systematic review design.

### Study selection

The following databases were searched: MEDLINE, Embase and PsycInfo. The databases were searched in August 2021. No limitation on publication date was applied. Only studies available in English were included. The search strategy included groups of terms relating to personality disorder, psychosocial intervention and crisis. The full search strategy can be found in the supplementary material available at https://doi.org/10.1192/bjo.2022.54. Existing reviews in the area were also hand-searched for relevant studies.^2,3^

Two reviewers worked independently on title and abstract screening. The full texts of all papers identified by the reviewers as potentially relevant were obtained and screened. All eligible full-text studies were subjected to forward and backward citation tracking to identify any additional relevant studies.

### Data extraction

Data were extracted into predefined tables by L.N. The data extracted comprised study characteristics (study design, sampling methods, study setting, eligibility criteria, outcome measures utilised), participants’ characteristics (age, gender, ethnicity, diagnosis), intervention characteristics (intervention aims; session number, length and content; modality; therapist profession; and comparator intervention type), data analysis and data on the outcomes examining efficacy of the intervention.

### Risk of bias assessment

Quality assessment was undertaken using the relevant Cochrane risk of bias assessment tool: the risk of bias tool for randomised trials (RoB 2), risk of bias tool for cluster randomised trials (cluster RoB 2) or risk of bias in non-randomised studies of intervention (ROBINS-I). All tools broadly examine the risk of bias in the following domains: selection bias, performance bias, attrition bias, detection bias and reporting bias.^16^ The specific ratings for each category for each tool can be found in the supplementary material.

### Data analysis

Given the limited number of identified studies and diversity in study design, we undertook a narrative synthesis.^17^ Narrative synthesis offers a framework for the synthesis of studies in such circumstances. Narrative synthesis involves four key elements: developing a theory of how the intervention works, why and for whom; developing a preliminary synthesis of findings of included studies; exploring relationships in data; and assessing the robustness of the synthesis. Initially, the study and individual participant characteristics were narratively summarised. We then compared the types of interventions utilised and examined their characteristics. We then examined the types of outcome measure used to examine the impact of the interventions. Finally, we narratively described the efficacy of the interventions. Owing to the paucity of studies and diversity in outcomes we were unable to undertake any statistical meta- analysis.

## Results

**Fig. 1:**
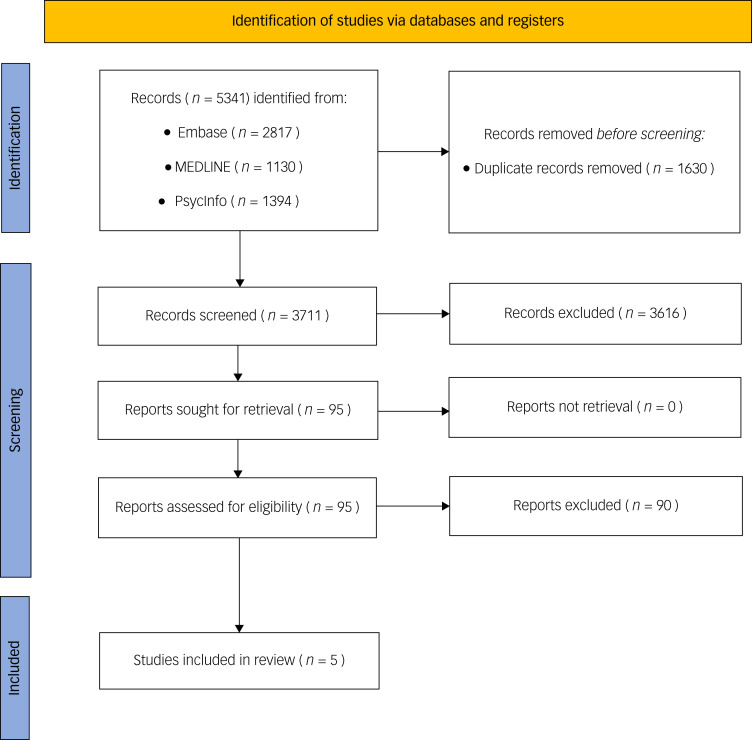
PRISMA flow diagram.

A total of 5431 studies were initially identified. After the removal of duplicates (*n* = 1630), the remaining 3711 studies were examined at title and abstract level. Of these, 95 papers were sourced for their full texts, 90 of which were excluded as they did not meet the review's inclusion criteria (see the outlined rationale in the supplementary material), leaving 5 studies which were included in the review (Fig. 1). Existing reviews were also hand-searched for additional references but no further eligible studies were identified. Two of the five studies were reports from the same trial, so four individual trials were included in the review.

### Study characteristics

The study characteristics are outlined in Table 1. Two studies were randomised controlled trials (RCTs),^18,19^ one study was a cluster RCT^20,21^ and one was a non-randomised controlled trial.^22^ All studies were conducted in Western countries: two in the UK,^18,19^ one in Australia^20^ and one in the USA.^22^ Two of the studies were undertaken in crisis services (one community and one in-patient),^18,22^ whereas the other two were undertaken in community mental health services. All the studies were relatively small (range *n* = 20–88) except for the cluster RCT, which had a sample of *n* = 642. The most common diagnoses that participants had across the studies were BPD and EUPD. One study^22^ also included a small sample of participants (*n* = 4) with a diagnosis of post-traumatic stress disorder (PTSD). The majority of participants were White and female.

**Table 1:** Study characteristics

Study	Design	Inclusion criteria	Study setting	Sample size	Participants’ demographics	Intervention	Comparison	Outcome measures
Grenyer et al, 2018;^20^ Huxley et al, 2019^21,^a^^	Cluster RCT	(a) Aged 12 years or over; (b) at least one in-patient admission within previous 18 months; (c) a primary diagnosis of personality disorder based on ICD-10	Crisis mental health service, New South Wales, Australia	EC: *n* = 335 TAU: *n* = 307 Total: *n* = 642 Total for the secondary analysis: *n* = 191	Age (mean 36.8; range 14–90); gender (male *n* = 318; female *n* = 324); diagnosis (BPD *n* = 642)	Stepped-care model of crisis psychiatric management: three or four 50 min sessions of crisis-focused therapy over 1 month. Aim: crisis management	TAU	Routinely data only Primary outcomes: readmission (number of bed-days and number of admissions) Secondary outcomes: number of A&E presentations
Davidson et al, 2014^18^	Feasibility RCT	(a) Aged 18–65 years; (b) scoring scored ≥3 on the SAPAS; (c) presence of personality disorder based on the SCID-II	Psychiatric liaison service, Glasgow, UK	EC: *n* = 14 TAU: *n* = 6 Total: *n* = 20	Diagnosis of simple personality disorder (*n* = 4), diffuse personality disorder (*n* = 16), BPD (*n* = 17), avoidant personality disorder (*n* = 13), paranoid personality disorder (*n* = 8); *n* = 9 also met diagnostic criteria for substance misuse	Manual-assisted cognitive therapy: six sessions. Aim: reduce self-harm and distress and promote engagement with services	TAU	Researcher collected data only Outcomes: suicide (BSS); anxiety and depression (HADS); alcohol use (AUDIT)
Borschmann et al, 2013^19^	Feasibility RCT	(a) Aged 18 years or older; (b) met diagnostic criteria for borderline personality disorder using the SCID-II borderline personality disorder subsection; (c) self-harmed in the previous 12 months; (d) under the care of a CMHT; (e) capacity to consent to participation	CMHTs, London, UK	EC: *n* = 46 TAU: *n* = 42 Total: *n* = 88	Age (mean 35.8); gender (male *n* = 17; female *n* = 71); ethnicity (White British *n* = 65; Other *n* = 23); diagnosis (BPD *n* = 88)	Joint crisis planning intervention: single 60 min session. Aim: crisis plan development	TAU	Combined routine and researcher collected data Outcomes: self-harm (study questionnaire items), anxiety and depression (HADS); therapeutic alliance (WAI), patient satisfaction (CSQ); service engagement (SES); well-being (WEMWBS);^b^ social functioning (WSAS); treatment experience (TES); quality of life (EQ-5D); resource use (AD-SUS); psychiatric admissions (routine data). Primary outcome measure: self-harming behaviour occurrence
Laddis, 2010^22^	Non-RCT	(a) 19–65 years of age; (b) diagnosed with BPD or PTSD based on screening using a structured interview; (c) in behavioural crisis	Crisis stabilisation Unit, Massachusetts, USA	EC: *n* = 32 TAU: *n* = 26 Total: *n* = 58	Age (mean 35.2); gender (male *n* = 9; female *n* = 49); diagnosis (BPD *n* = 54; PTSD *n* = 4)	Cape Cod Model: intervention is brief but session number unclear. Aim: improve caregiving relationships to reduce crisis	TAU	Combined routine and researcher collected data Measures: BPRS, BSI, CO,^c^ CSO^d^ and list of medications Primary outcomes: BPRS Secondary outcomes: CO, CSO

A&E, Accident and Emergency department; AD-SUS, Adult Service Use Schedule; AUDIT, Alcohol Use Disorders Identification Test; BPD, Bipolar Disorder; BPRS, Brief Psychiatric Rating Scale; BSI, Brief Symptom Inventory; BSS, Beck Scale for Suicide Ideation; CMHT, Community Mental Health Team; CO, Client Observation; CSO, Client Self-Observation; CSQ, Client Satisfaction Questionnaire; EC, Experimental Condition; EQ-5D, EuroQol quality of life measure; HADS, Hospital Anxiety and Depression Scale; PTSD, Post-Traumatic Stress Disorder; RCT, Randomised Controlled Trial; SAPAS, Standardised Assessment of Personality Abbreviated Scale; SCID-II, Structured Clinical Interview for DSM-IV Axis II Personality Disorders; SES, Service Engagement Scale; TAU, treatment as usual; TES, Treatment Experience Scale; WAI, Working Alliance Inventory; WEMWBS, Warwick–Edinburgh Mental Wellbeing Scales; WSAS, Work and Social Adjustment Scale.

a. Huxley et al^21^ is a secondary analysis of a service. It comprised two studies: study 1, examination of referral pathways and intervention retention; study 2, examination of symptom change during a brief intervention. Only study 1 data were eligible for inclusion in this review. Hereafter, we only cite the primary paper of Greyner et al^20^.

b. Added to the battery, on the advice of the project advisory group, after 48 participants had entered the study. Therefore, data are available for only 40 participants (45.4%) at baseline.

c. This is a pilot rating scale developed by the study's author. It consists of five items of observable behaviour that are characteristic of behavioural crises in BPD and PTSD. It was completed by a member of nursing staff after treatment and included both a retrospective pre-treatment and a follow-up rating.

d. This is a pilot rating scale developed by the study's author which focuses on psychological events that underpin observable behaviour found in the Client Observation measure. It consists of nine items. A structured interview with research staff provided well-differentiated markers for the patient's self-ratings. It was administered after treatment and included both a retrospective pre-treatment and a follow-up rating.

### Risk of bias

**Fig. 2:**
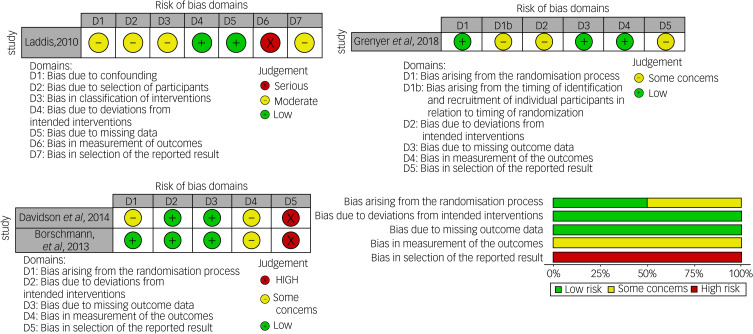
Risk of bias assessment. For Davidson et al,^18^ Borschmann et al^19^ and Grenyer et al,^20^ domains D1 and D1b are selection bias, D2 is performance bias, D3 is attrition bias, D4 is detection bias and D5 is selective reporting bias. For Laddis,^22^ D1 and D2 are selection bias, D3 and D4 are performance bias, D5 is attrition bias, D6 is detection bias and D7 is selective reporting bias.

Figure 2 summarises the risk of bias for the included trials. The detailed risk of bias assessment can be found in the supplementary material. Overall, the included studies demonstrated moderate risk of bias.

Selection bias refers to the systematic differences between baseline characteristics of the groups that are compared, which has been found to inflate treatment effect sizes.^23,24^ Two studies^19,20^ demonstrated low risk of bias in this domain, as their randomisation and allocation concealment strategies were clearly explained and minimised this bias, for example by having a computerised randomisation system and independent individual undertaking randomisation, and one study^18^ had some concerns due to a lack of detailed reporting on randomisation strategies. The final study^22^ had a moderate risk of bias as participants were not randomised to the study arms and therefore bias was likely to be present in the group allocations.

Performance bias is the systematic differences between groups in the care that is provided. All studies had some concerns or moderate risk in this area as participants knew their treatment allocation, which is the case psychological therapy trials.^25^

Attrition bias is the systematic differences between groups in withdrawals from a study. Grenyer et al^20^ had low risk of bias as no participants were lost to follow-up because the primary outcome was routinely collected data. Laddis^22^ had low attrition rates (97% of participants completed the outcomes) and was rated as low risk of bias. The remaining two studies showed some concerns because attrition rates were between 17 and 30% overall, but both studies also completed an intention-to-treat analysis to minimise bias.^18,19^

Detection bias refers to systemic disparities in how outcomes are measured between groups, and masking (‘blinding’) of outcome assessors is generally used to lessen the likelihood of this type of bias. Two studies were at low risk of bias as they had masked outcome assessors.^18,19^ Grenyer et al^20^ was also low risk of bias as they used routinely collected data for their primary outcome, which minimises this type of bias. The remaining study was at high risk of bias as raters were not masked.^22^

Finally, reporting bias, defined as when the dissemination of research findings is influenced by the nature and direction of results, was shown to be high risk for two studies as they lacked pre-specified published protocols.^18,19^ Another included study scored as ‘some concerns of bias’,^20^ as the article linked to a protocol from 2011, but this was in the form of a ward document not a published protocol. The fourth study scored as ‘moderate risk’ of bias as there were being multiple analyses of the intervention–outcome relationship.^22^

### Characteristics of interventions used

#### Control conditions

All four studies used treatment as usual (TAU) as their control condition. For three studies, TAU included access to community mental health teams, routine care plans, psychiatrists and mental health nurses. Access to crisis services was included in TAU in only one study.^22^

#### Experimental conditions

All four studies utilised different approaches to crisis intervention. Three utilised a psychological therapy (one used cognitive therapy, one was unspecified and one used the Cape Cod model) and one used a crisis planning intervention. However, all were consistent in offering brief interventions (range 1–6). All studies also included a focus on crisis planning and crisis management. Grenyer et al^20^ examined a three-stage stepped-care model of crisis intervention. This included the triaging of participants, brief manualised psychological therapy focusing on crisis management, safety care planning, symptom reduction and improving psychosocial functioning, alongside TAU. Davidson et al^18^ offered a six-session cognitive therapy intervention delivered by a clinical psychologist and psychiatrist, which focused on addressing self-harm behaviour, reducing distress and promoting engagement in services. Borschmann et al^19^ offered a single-session crisis planning intervention with the aim of developing a crisis plan that was distributed to the care team and uploaded to the participant's medical records. Finally, Laddis^22^ offered a intervention based on the Cape Cod model,^26^ which is a gestalt-based process-oriented therapy aiming to cultivate an optimistic approach to relationships. The intervention aims to identify the individual's main caregiving relationships and explore ways to improve trust and strengthen the relationship.

### Examined outcomes

The four studies utilised diverse outcome sets to examine the feasibility or effectiveness of their intervention. One study^20^ solely used routinely collected data, one^18^ solely used researcher-collected data and two^19,22^ used a combination of both. The most frequently measured outcome was psychiatric symptoms, which were examined by three studies.^18,19,22^ Two studies^18,19^ utilised the Hospital Anxiety and Depression Scale (HADS)^27^ to measure depression and anxiety and one study^22^ used multiple measures to examine overall psychiatric symptoms (Brief Psychiatric Rating Scale (BPRS);^28^ Brief Symptom Inventory (BSI);^29^ and patient- and staff-observed self-report measures of BPD and PTSD). Two studies^19,20^ examined rates of hospital readmission using routinely collected data. The remaining outcomes were measured only once across the studies. These included outcomes of self-harm, suicidal ideation, alcohol use, therapeutic alliance, well-being, patient satisfaction with services, service engagement, social functioning, treatment experience, quality of life and resource use.

### Summary of findings across studies

Overall, the included studies incorporated data on feasibility, acceptability and efficacy of the interventions. One study (Borschmann et al^19^) reported on the feasibility and acceptability of the crisis-focused psychosocial intervention. Its authors deemed recruitment (88 participants recruited across 17 months) and baseline (100% of measures complete) and follow-up (71.6–82.9% of measures complete) assessment measure completion as feasible. Their intervention (joint crisis plans, JCPs) was also deemed feasible as they found that 89.1% participants attended the JCP meeting, 73.5 and 44.1% of participants used their JCP during and outside a crisis respectively, 56.1% agreed for a copy to be uploaded to their medical records, 47.1% described ‘a greater feeling of control’ over their problems and 85.2% would recommend JCPs to other patients.

The rest of the findings across the literature pertained to the efficacy of the interventions, showing mixed results. The available means for the reported outcomes are given in the supplementary material. Borschmann et al's JCP intervention found no significant difference between groups in the proportion (OR = 1.9, 95% CI 0.53–6.5, *P* = 0.33) or number (RR = 0.74, 95% CI 0.34–1.63, *P* = 0.46) of self-harm acts at 6-month follow-up (primary outcome).^19^ When looking at secondary outcomes no significant differences were identified at follow-up (working alliance, patient satisfaction, well-being, anxiety, depression, self-esteem and therapeutic alliance). Davidson et al^18^ found significantly lower scores on the measure of suicidality (Beck Scale for Suicide Ideation; *t*(11.18) = −3.64, *P* = 0.004) and anxiety and depression (HADS; *t*(12.4) = −3.68, *P* = 0.003) in the intervention group compared with TAU. No difference was found in number of self-harm episodes, alcohol use (Alcohol Use Disorders Identification Test) or service use. Grenyer et al^20^ found that their crisis intervention significantly reduced the number of bed-days for the crisis intervention group compared with TAU (*F*(1,640) = 4.301, *P* = 0.038) but not the number of admissions (*F*(1,640) = 0.006, *P* = 0.937). They also found that participants in the intervention group were 1.28 times more likely to not present at emergency departments than TAU participants (95% CI 1.17–1.40, χ^2^ = 19.980, *P* = 0.000). Laddis^22^ found a significant improvement in participants’ symptoms (total score on the BPRS; *R*^2^ = 0.402, F change 27.70, *P* ≤ 0.001) following their intervention, but this finding was not replicated with the BSI (self-report measure of symptoms; *F* = 1.031, *P* = 0.314, partial η^2^ = 0.018.). The Client Observations, a staff-rated measure of behavioural characteristics of BPD (mean = 7.0, *F* = 11.859, *P* = 0.001, partial η^2^ = 0.180), and the Client Self-Report Observations, a patient-rated measure of behavioural characteristics of BPD (*F* = 6.246, *P* = 0.016, partial η^2^ = 0.104), both showed a significant difference favouring the crisis intervention. Differences were found between groups in relation to medication use: the number of medications prescribed was significantly higher in the control group compared with the intervention group (*P* = 0.01).^22^ We were unable to examine the outcomes of function and quality of life as planned owing to a lack of data in primary studies.

## Discussion

This systematic review aimed to examine the current evidence base pertaining to crisis-focused psychosocial interventions for people with a diagnosis of BPD. The review identified five studies reporting on four trials that examined such interventions. This is an improvement on the Borschmann et al review^3^ examining the same subject, which identified only two incomplete trials. The present review was able to demonstrate that the crisis psychosocial interventions developed to date are generally feasible and acceptable to people with BPD and that they have some potential impact on outcomes. However, findings should be interpreted tentatively owing to the limited literature identified.

Three interventions were delivered in community settings and one was delivered in a crisis stabilisation unit. This review demonstrates that there is still a paucity of literature examining crisis-focused psychosocial interventions for people with a diagnosis of BPD, especially in-patient-based interventions, and that more research is needed to develop the evidence base. The interventions included were quite broad in their approach to crisis management, although some key components can be identified. All the interventions included some form of crisis/safety plan to help the person prevent future crises.^18–20,22^ Crisis or safety plans are plans that include the identification of early warning signs of crisis, identification of sources of support and coping strategies during a crisis, and identification of how the person wants to be treated if they were to be treated again by crisis or in-patient services.^19^ Moreover, two of the interventions included coping strategy development to help the person cope with future crises. In one study this drew on cognitive–behavioural therapy^18^ and in the other it drew on dialectical behaviour therapy.^20^ Two studies also focused on engagement with the wider team: one outlined that the intervention aimed to promote engagement^20^ and the other outlined the sharing of a crisis plan with the wider team.^19^ One study^22^ focused on developing the individual's interpersonal relationships in hope that this may prevent future crises. This may suggest that these components of an intervention may be helpful when considering implementing interventions with people diagnosed with BPD in crisis.

This review identified that there was limited consensus on the types of outcome measure utilised to measure the usefulness of the interventions. The only outcomes used by more than one study were anxiety and depression, and hospital readmission. It seems reasonable to conclude that measuring such outcomes may be helpful in future trials in this area. Suicide,^18^ self-harm^19^ and presentation to crisis services^20^ may also be useful outcomes, which were each measured by one study. In intervention research, there is general consensus that a core outcome set, i.e. a recommended set of outcomes to evaluate specific health interventions, is best practice for effectively evaluating outcome and developing the evidence base.^30^ There also appears to be a need to develop a core outcome set for crisis-focused psychosocial interventions for personality disorder alongside the development of the evidence base for such interventions.

### Strengths and limitations

This review had a number of strengths. It followed rigorous guidelines in terms of the undertaking of systematic review. We followed the PRISMA guidelines,^31^ pre-registered the systematic review protocol and transparently followed the narrative synthesis guidelines to inform the analysis,^17^ which are all best practice. We also undertook a review in a field in which research is underdeveloped. Although only a limited number of studies were identified, we have been able to summarise available literature and make some tentative recommendations for current practice and future research in this field.

In terms of limitations, this study did deviate from the initial protocol. First, in the protocol we cited the older version of the risk of bias tool but we used the newer one in the final review. We also used the Cochrane risk of bias tool for cluster randomised trials (cluster RoB 2) and the tool for non-randomised studies (ROBINS-I), which were the appropriate tools for the relevant study designs but were not described in our protocol. Another deviation was using three databases rather than four. This was because the fourth (the Cochrane Central Register of Controlled Trials, CENTRAL) predominantly draws on one of the already included databases (Embase), which meant a high likelihood of duplication. Another limitation was the paucity of studies identified. This meant that were unable to undertake any meta-analysis to understand the efficacy of the intervention of interest. Our search strategy was limited to the terms personality disorder, complex trauma and complex emotional difficulty. Although this is broad enough to include people who have presentations that align with a personality disorder, we did not include the terms complex PTSD or complex emotional needs, which are used quite frequently in more recent literature.^10^ This may have led to the omission of some studies and should be rectified in future reviews.

### Future research

There is a significant need to improve the evidence base for crisis-focused psychosocial interventions for people with a diagnosis of personality disorder and a large definitive trial is required. The qualitative literature demonstrates that there is great dissatisfaction from patients regarding crisis services so an intervention co-produced with patients is essential.

## Data Availability

Data availability is not applicable to this article as no new data were created or analysed in this study.

## Supplementary material

For supplementary material accompanying this paper visit http://doi.org/10.1192/bjo.2022.54. click here to view supplementary material
